# A prospective observational prevalence study of elevated HbA1c among elective surgical patients

**DOI:** 10.1038/s41598-020-76105-2

**Published:** 2020-11-04

**Authors:** L. M. Teo, W. Y. Lim, Y. Ke, I. K. L. Sia, C. H. Gui, H. R. Abdullah

**Affiliations:** 1 Division of Anaesthesiology and Perioperative Medicine, Sengkang General Hospital, Singapore General Hospital, Outram Road, Singapore, 169608 Singapore; 2 Singhealth Anaesthesiology Residency Program, Division of Anaesthesiology and Perioperative Medicine, Singapore General Hospital, Outram Road, Singapore, 169608 Singapore; 3 Duke-NUS (National University of Singapore) Medical School, 8 College Rd, Singapore, 169857 Singapore; 4 National University of Singapore, Yong Loo Lin School of Medicine, 1E Kent Ridge Road, Singapore, 119228 Singapore

**Keywords:** Biomarkers, Risk factors

## Abstract

Type 2 Diabetes Mellitus (DM) is a chronic disease with high prevalence worldwide. Using glycated haemoglobin (HbA1c) as a surrogate for potential pre-DM and DM conditions, our primary objective was to determine the HbA1c epidemiology in non-cardiac elective surgical patients in Singapore. Our secondary aim was to identify risk factors associated with elevated HbA1c. We conducted a prospective, observational single-centre study in adult patients. HbA1c screening was performed. Patient demographics and comorbidities were recorded. Patients were divided into those with HbA1C ≤ 6.0% and HbA1C ≥ 6.1%. Regression analyses were performed to identify associated factors. Subgroup analysis was performed comparing patients with HbA1C ≥ 6.1% and HbA1C ≥ 8.0%. Of the 875 patients recruited, 182 (20.8%) had HbA1c ≥ 6.1%, of which 32 (3.7%) had HbA1c ≥ 8%. HbA1C ≥ 6.1% was associated with Indian ethnicity [1.07 (1.01–1.13), p = 0.023], BMI > 27.5 [1.07 (1.02–1.11), p = 0.002], higher preoperative random serum glucose [1.03 (1.02–1.04), p < 0.001], pre-existing diagnosis of DM [1.85 (1.75–1.96), p < 0.001] and prediabetes [1.44 (1.24–1.67), p < 0.001], and peripheral vascular disease [1.30 (1.10–1.54), p = 0.002]. HbA1c ≥ 8% had an additional association with age > 60 years [0.96 (0.93–0.99), p = 0.017]. The prevalence of elevated HbA1c is high among the surgical population. Targeted preoperative HbA1c screening for at-risk elective surgical patients reduces cost, allowing focused use of healthcare resources.

## Introduction

Type 2 Diabetes Mellitus (DM) is a chronic disease with high prevalence worldwide. The International Diabetes Federation (IDF) estimates that today, 463 million people (9.3% of adults between 20 and 79 years old) worldwide have diabetes. By 2045, this number is predicted to reach 700 million^1^. DM imposes a huge health burden on society. The World Health Organization (WHO)^2^ estimated that 1.6 million deaths were directly attributable to DM in 2016. In addition to increasing the risk of vascular diseases^3^ by two-fold, DM is a major cause of limb amputations^4^, blindness^5^ and renal failure^6^. This indirectly translates to a high economic burden due to hospitalisations, Emergency Department visits, outpatient physician visits, medications, laboratory tests and allied health services^7^. According to the IDF’s estimation, the annual global health expenditure on DM is USD $760 billion and is expected to grow with increasing prevalence of the disease.

One of the strategies for battling this epidemic is early screening and detection of DM. In addition to fasting blood glucose (FBG), the WHO^8^ and the American Diabetes Association (ADA)^9^ recommend that glycated haemoglobin (HbA1c) be used as a diagnostic test, with a cut off value of 6.5% for the diagnosis of DM and 6.1% for the diagnosis of prediabetes. Since March 2019, HbA1c has been endorsed by the Ministry of Health, Singapore, as an alternative screening test for DM^10^. The perioperative period presents a good opportunity to screen the adult surgical population for DM. These patients are a “captive audience” who have chosen to present themselves to a healthcare facility. Furthermore, blood investigations are routinely performed pre-operatively and opportunistic DM screening can be performed. Globally, HbA1c screening for DM in elective surgical patients has been initiated since 2013^11–14^.

The perioperative period also provides an opportunity to identify patients with prediabetes and newly diagnosed DM, with appropriate counselling and initiation of treatment of patients with newly diagnosed prediabetes and DM. For known diabetics, this period constitutes a checkpoint for the control of the chronic condition. DM-associated complications (e.g. microvascular and macrovascular) may also affect surgical outcomes^9,15^ adversely. Poorly controlled DM, represented by elevated HbA1C, further exacerbates this as persistent hyperglycemia is a risk factor for endothelial dysfunction, post-operative sepsis, impaired wound healing and mortality^16–18^. Every 1% in HbA1c was associated with an increased likelihood of intensive care unit admission, hospital length of stay and greater risk of major complications^11^. As preoperative HbA1C has a significant impact on short- and long- term health outcomes, the perioperative period facilitates patient education and raises awareness. The importance of good glycaemic control should be emphasized to patients (both known and newly diagnosed) and optimization should be undertaken prior to surgery.

Currently, the epidemiology of elevated HbA1C levels among patients presenting for non-cardiac elective surgery in Singapore is not known. Using preoperative HbA1c and the established cut-off values^8,9^, our primary objective was to determine the proportion of patients with HbA1c ≥ 6.1% in elective, non-cardiac surgical patients in Singapore. Our secondary aim was to identify risk factors associated with HbA1c ≥ 6.1% and HbA1C ≥ 8.0%.

## Methodology

### Ethics approval

Ethics approval for the study was obtained from Singhealth’s Centralised Institutional Review Board (CIRB, Reference Number 2018/3225). This study is registered with the clinicaltrials.gov database (NCT04070963). The study protocol was performed in accordance to the Declaration of Helsinki.

### Study design

We conducted a prospective, observational single-centre study at the Preoperative Assessment Centre (PAC) of Singapore General Hospital (SGH) from May 2019 to Aug 2019. SGH is a 1700 bedded government-aided tertiary academic referral center that performs approximately 30,000 elective surgical procedures per annum. The inclusion criteria were: (1) adult patients aged 21 and above, and (2) non-cardiac elective operations who required preoperative blood investigations. We excluded patients who were unable to provide consent, or in situations where there was inadequate blood sample to perform the HbA1c test. Participants were approached and included in our study after written informed consent was obtained. Consent forms were in English, and participants from non-English speaking backgrounds were provided with a translator.

Patients’ demographic information including age, gender, ethnicity, and BMI were collected. The functional status, including METs and ASA status were recorded. Medical comorbidities including smoking history, DM, hypertension, dyslipidaemia, history of stroke, AMI or ischaemic heart disease, COPD were also recorded. Potential confounders, such as the presence of haemoglobinopathies e.g. thalassaemia were sought. These were sourced from our institution's clinical information system [Sunrise Clinical Manager (SCM), Allscripts, Illinois, USA] and stored in our enterprise data repository and analytics system (SingHealth-IHiS Electronic Health Intelligence System), which integrates information from multiple healthcare systems including administrative, clinical and ancillary healthcare systems. Medication history, in particular, the treatment of DM e.g. OHGA and insulin were reviewed. Surgical details such as the type of surgery and the associated surgical disciplines were also recorded.

HbA1c test was added to routine preoperative blood investigations for all participants. HbA1c measurement was carried out by immunoassay with the Roche Cobas c501 analyzer (Roche Diagnostics). Our method is accredited by the National Glycoprotein Standardization Program (NGSP) and standardized to the Diabetes Control and Complications Trial (DCCT) assay. We sought to determine the prevalence of elevated HbA1c (> 6.0%), with further stratification at the levels of HbA1c ≥ 8.0% among elective, non-cardiac surgical patients. This stratification is based on levels previously described in the literature^8,9,19^.

### Sample size estimation

Using a precision of 2% and published inpatient DM prevalence of 10%^20^ and 95% confidence interval (8–12% prevalence limit), the estimated sample size required was 865. A total of 888 patients were recruited, taking into consideration potential dropouts due to laboratory errors or insufficient blood samples for HbA1c testing.

### Statistical analysis

Patient demographics and clinical characteristics between the groups where HbA1C is ≥ 6.1% and ≤ 6.0% were compared (Table 1). HbA1c value of ≥ 6.1% was chosen based on local literature^21^. This group of patients is at risk of perioperative hyperglycaemia and poorer surgical outcomes^22,23^. For continuous variables, Kruskal–Wallis test was used for non-parametric variables and ANOVA was used for variables with normal distribution. For categorical variables, the chi-squared test was used to compare the proportions between the groups. Subgroup analysis for HbA1C ≥ 8.0% was done (Table 2).

**Table 1:** Patient demographics stratified by HbA1C cut-off of 6.0%.

	HbA1C ≤ 6.0% (N = 693)	HbA1C ≥ 6.1% (N = 182)	Total (N = 875)	p value	Missing
Age	49.6 (16.4)	61.6 (11.9)	52.1 (16.3)	< 0.001	
Gender (female)	346 (49.9)	74 (40.7)	420 (48.0)	0.026	
**Ethnicity**					
Chinese	497 (71.7)	116 (63.7%)	613 (70.1)	0.002	
Malay	75 (10.8)	23 (12.6%)	98 (11.2)		
Indian	68 (9.8)	35 (19.2%)	103 (11.8)		
Others	53 (7.6)	8 (4.4%)	61 (7.0)		
BMI	25.4 (5.2)	28.8 (5.9)	26.1 (5.5)	< 0.001	
ASA 1	159 (22.9)	3 (1.6)	162 (18.5)	< 0.001	
ASA 2	461 (66.5)	120 (65.9)	581 (66.4)		
ASA 3 and 4	73 (10.5)	59 (32.4)	132 (15.1)		
**Best known function**					
METs < 4	10 (1.5)	14 (8.4)	24 (2.9)	< 0.001	53
METs 4–10	177 (27.0)	43 (25.9)	220 (26.8)		
METs > 10	469 (71.5)	109 (65.7)	578 (70.3)		
**Admission type**					
Inpatient	503 (72.8)	142 (78.5)	645 (74.0)	0.122	3
Day surgery	188 (27.2)	39 (21.5)	227 (26.0)		
Hemoglobin (g dL^−1^)	13.7 (1.6)	13.4 (1.6)	13.6 (1.6)	0.006	
Preoperative Random Serum Glucose (mmol L^−1^)	5.5 (1.2)	8.4 (3.3)	6.1 (2.2)	< 0.001	
Creatinine (μmol L^-1^)	76.2 (71.4)	106.1 (122.3)	82.4 (85.3)	< 0.001	
HbA1C value (%)	5.4 (0.4)	7.2 (1.2)	5.74 (0.97)	< 0.001	
Smoker	66 (9.5)	14 (7.7)	80 (9.1)	0.743	
**Nature of operation**					
General Surgery	194 (28.0)	45 (24.7)	239 (27.3)	0.378	
Urology	85 (12.3)	35 (19.2)	120 (13.7)	0.015	
Gynecology	74 (10.7)	9 (4.9)	83 (9.5)	0.019	
Vascular	18 (2.6)	20 (11.0)	38 (4.3)	< 0.001	
Orthopaedics	233 (33.6)	58 (31.9)	291 (33.3)	0.655	
Subspecialty surgeries	110 (15.9)	20 (11.0)	130 (14.9)	0.099	
**Past medical history**					
Preexisting diabetes mellitus	31 (4.5)	136 (74.7)	167 (19.1)	< 0.001	
Pre-diabetes	6 (0.9)	9 (4.9)	15 (1.7)	< 0.001	
Hypertension	167 (24.1)	128 (70.3)	295 (33.7)	< 0.001	
Previous stroke	11 (1.6%)	18 (9.9%)	29 (3.3%)	< 0.001	
Previous AMI	17 (2.5%)	26 (14.3%)	43 (4.9%)	< 0.001	
Peripheral vascular disease	1 (0.1%)	12 (6.6%)	13 (1.5%)	< 0.001	
COPD	4 (0.6%)	4 (2.2%)	8 (0.9%)	0.041	

Values are mean (SD) or number (proportions).

Mann Whitney U test for continuous variable and Chi Square Test—for discrete variable. Mean or count (± SD or %).

*BMI* Body Mass Index, *ASA* American society of Anaesthesiologists physical status classification, *MET* metabolic equivalents, *OHGA* Oral Hypoglycemic agents, *AMI* Acute myocardial infarction, *COPD* Chronic obstructive pulmonary disease, *ACE* Angiotensin-Converting Enzyme, *ARB* Angiotensin II receptor blocker.

**Table 2:** Univariable and Multivariable analysis of those significant for HbA1C ≥ 6.1%.

	HbA1C ≥ 6.1%			HbA1C ≥ 8.0%		
	Univariable	Multivariable		Univariable	Multivariable	
	p value	p value	OR (95% Cl)	p value	p value	OR (95% Cl)
**Age**						
< 40	Reference	0.723	1.01 (0.96–1.06)	Reference	0.965	1.00 (0.97–1.03)
40–60	< 0.001	0.370	1.03 (0.97–1.09)	0.068	0.017	0.96 (0.93–0.99)
> 60	< 0.001			0.339		
Gender (female)	0.0259	0.557	0.99 (0.95–1.03)	0.492		
**Race**						
Chinese	Reference	0.863	1.01 (0.95–1.07)	Reference	0.308	1.00 (0.98–1.06)
Malay	0.300	0.023	1.07 (1.01–1.13)	0.054	0.003	1.10 (1.02- 1.09)
Indian	< 0.001	0.290	0.96 (0.90–1.03)	< 0.001	0.585	0.99 (0.95–1.03)
Others	0.284			0.353		
BMI 18.5—27.5	Reference			Reference		
BMI < 18.5	0.043	0.378	0.96 (0.87–1.05)	0.569	0.884	1.00 (0.95–1.07)
BMI > 27.5	< 0.001	0.002	1.07 (1.02–1.11)	< 0.001	0.222	1.02 (0.99–1.04)
ASA 1	Reference			Reference		
ASA 2	< 0.001	0.778	1.01 (0.96–1.06)	0.057	0.616	1.01 (0.98–1.04)
ASA 3 and 4	< 0.001	0.749	1.01 (0.94–1.09)	< 0.001	0.725	1.01 (0.97–1.05)
MET 1–4	Reference			Reference		
MET 4–10	< 0.001	0.129	1.10 (0.97–1.24)	0.151		
MET > 10	< 0.001	0.733	1.02 (0.91–1.14)	0.198		
**Hemoglobin (g/dL)**						
> 13	Reference	0.599	1.01 (0.97–1.06)	Reference	0.428	1.01 (0.99–1.04)
11–13	0.009	0.130	0.93 (0.85–1.02)	0.031	0.002	0.92 (0.88–0.97)
< 11	0.590			0.339		
Preoperative random serum glucose (mmol/L)	< 0.001	< 0.001	1.03 (1.02–1.04)	< 0.001	< 0.001	1.03 (1.02–1.04)
Creatinine	< 0.001	0.860	1.00 (1.00–1.00)	0.393		
Diabetes mellitus	< 0.001	< 0.001	1.85 (1.75–1.96)	< 0.001	< 0.001	1.09 (1.05–1.13)
Prediabetes	< 0.001	< 0.001	1.44 (1.24–1.67)	0.455		
Hypertension	< 0.001	0.094	1.04 (0.99–1.09)	< 0.001	0.069	0.97 (0.95- 1.00)
Previous stroke	< 0.001	0.553	0.97 (0.87–1.08)	< 0.001	0.150	1.05 (0.99–1.12)
Previous AMI	< 0.001	0.301	1.05 (0.96–1.15)	< 0.001	0.315	1.03 (0.98–1.09)
Peripheral vascular disease	< 0.001	0.002	1.30 (1.10–1.54)	< 0.001	0.016	1.13 (1.03–1.24)
COPD	0.041	0.422	0.92 (0.76–1.12)	0.169		

*BMI* Body Mass Index, *ASA* American society of Anesthesiologists physical status classification, *MET* metabolic equivalents, *AMI* Acute myocardial infarction, *COPD* Chronic obstructive pulmonary disease.

Multivariable logistic regression was performed to determine the independent predictors for HbA1C ≥ 6.1%. Variables with known clinical probability for contributing to elevated HbA1c such as demographics and preoperative clinical risk factors, as well as covariables with significance levels of p < 0.1 in the univariate analysis were also included in the multivariable model. The effect size was reported as an odds ratio (OR) and its 95% confidence interval (CI). To avoid multicollinearity, Variance Inflation Factor (VIF) was used to ensure all factors in the regression models do not exceed 5.0^24^.

Preoperative serum glucose was further assessed for its correlation and prediction of preoperative HbA1C value. Correlation between haemoglobin level and HbA1c was investigated via Pearson correlation coefficient. All analyses, statistical computing and visualisation were carried out in the R environment version 1.2.1335 using “ggplot2” R library package^25^.

## Results

A total of 888 patients were recruited from 9 May 2019 to 27 Aug 2019 in the preoperative assessment centre (PAC) of a tertiary hospital in Singapore. Of those, 13 were excluded as they had a history of thalassemia. Our final analysis was carried out with 875 patient observations.

**Figure 1:**
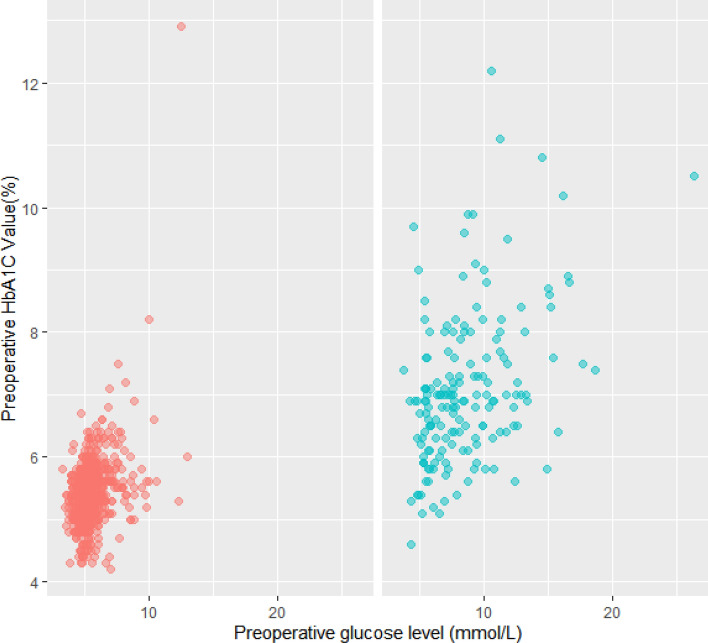
Comparison of preoperative HbA1c and glucose level in patients with DM (*blue*) and without DM (*red*). Each dot represents a single patient. *HbA1c* glycated haemoglobin, *DM* diabetes mellitus.

Stratifying patients based on HbA1c, 693 (79.2%) had HbA1C ≤ 6.0% and 182 (20.8%) patients who had HbA1c ≥ 6.1%. The mean age was 49.6 in the HbA1C ≤ 6.0% group and 61.6 in the HbA1C ≥ 6.1% group. The prevalence of pre-existing diabetes was 31 (3.5%) in the HbA1C ≤ 6.0% group and 136 (15.5%) in the HbA1C ≥ 6.1% group. Of note, 37 (4.2%) patients who did not have a diagnosis of DM or Pre-DM had HbA1C of ≥ 6.1%. The HbA1C ≥ 6.1% group also had more males, more likely to be Indian ethnicity, metabolic equivalents (METs) < 4, higher American Society of Anaesthesiologist (ASA) status, higher body mass index (BMI), higher preoperative random serum glucose and creatinine levels and higher incidence of chronic diseases such as hypertension, previous stroke or acute myocardial infarction (AMI), chronic obstructive pulmonary disease (COPD) and peripheral vascular disease (Table 1). Figure 1 shows the relationship between HbA1C and preoperative glucose levels as stratified by the presence or absence of known DM.

Based on electronic medical records, 167 (19.1%) of the patients are known diabetics and 15 (1.7%) had a pre-existing diagnosis of prediabetes. Amongst the 693 (79.2%) patients who were not known to have DM, 5 patients had HbA1c ≥ 6.5%, resulting in a prevalence of undiagnosed DM of 0.5%. There were 32 (3.7%) patients who were not known to have prediabetes.

Adjusted regression analysis showed that HbA1C of ≥ 6.1% was associated with Indian ethnicity [1.07 (1.01–1.13), p = 0.023] [(OR(95%Cl), p value)], BMI more than 27.5 [1.07 (1.02–1.11), p = 0.002], higher preoperative random serum glucose (mmol/L) [1.03 (1.02–1.04), p < 0.001], pre-existing diagnosis of DM [1.85 (1.75–1.96), p < 0.001] and prediabetes [1.44 (1.24–1.67), p < 0.001], and diagnosis of peripheral vascular disease [1.30 (1.10–1.54), p = 0.002]. The adjusted analysis did not detect significance in age, ASA status, MET functional status, preoperative haemoglobin and creatinine levels, and other chronic diseases such as hypertension, COPD and previous stroke and AMI (Table 2).

Subgroup analysis of HbA1C ≥ 8.0% was conducted for poorly controlled diabetics. 32/875 (3.7%) had HbA1C ≥ 8.0%. Factors that are associated with HbA1C ≥ 8.0% include age of > 60 years old [0.96 (0.93–0.99), p = 0.017], Indian ethnicity [1.10 (1.02–1.09), p = 0.003], higher preoperative random serum glucose level [1.03 (1.02–1.04), p < 0.001], pre-existing diagnosis of DM (1.09 (1.05–1.13), p < 0.001), and peripheral vascular disease [1.13 (1.03–1.24), p = 0.016]. Interestingly, haemoglobin of < 11.0 g/dL was associated with lower incidence of HbA1C > 8.0% on the adjusted analysis [0.92 (0.88–0.97), p = 0.002] (Table 2).

Within the group of 167 diagnosed diabetics, those who were on insulin with or without oral hypoglycaemic agent (OHGA) account for 12.6% of the patients. Table 3 shows the distribution of the patients on DM medication and their HbA1c levels. There were 109 (65.2%) patients on metformin, 54 (32.3%) patients on sulphonylurea and 31 (18.5%) patients on sodium-glucose cotransporter 2 (SGLT2) inhibitors.

**Table 3:** Medications in those diagnosed with DM.

	HbA1C ≤ 6.0%(N = 31)	HbA1C ≥ 6.1% (N = 136)	Total (N = 167)	p value
**Treatment of diabetes**				< 0.001
OHGA	15 (48.4%)	102 (75.0%)	117 (70.1%)	
Insulin	2 (6.5%)	4 (2.9%)	6 (3.6%)	
OHGA + insulin	0 (0.0%)	15 (11.0%)	15 (9.0%)	
Diet	14 (45.2%)	15 (11.0%)	29 (17.4%)	

Values are number (proportions).

*DM* diabetes mellitus, *OHGA* oral hypoglycemic agents.

## Discussion

20.8% of our study population had HbA1c ≥ 6.1%. Within this group, 37 (4.2%) patients did not have a diagnosis of pre-DM or DM, suggesting that we could utilize the perioperative encounter as a screening opportunity. However, in our institution, the cost of HbA1c testing is 2.5 times that of blood glucose testing and routine preoperative HbA1c screening for all patients may not be cost-effective. As such, we recommend a more targeted approach. Based on our analysis of risk factors, HbA1c screening should be considered in patients aged 60 and above, of Indian ethnicity, have a high BMI > 27.5 or have pre-existing DM, prediabetes or peripheral vascular disease. For patients with HbA1c between 6 and 8%, close monitoring of blood glucose levels four times a day^26,27^ in the postoperative period to achieve blood glucose levels between 6 and 10 mmol/L is recommended^26,28^.

Diabetes mellitus increases the risk for perioperative complications such as wound infection (OR 2.3)^29^, acute kidney injury (OR 4.15)^30^, prolonged hospitalization (OR 1.60)^30^ and even mortality (OR 1.51)^31^. Every 1% in HbA1c is also associated with an increased likelihood of ICU admission, hospital LOS and greater risk of major complications^11^. Elective surgery should be deferred, if possible, in patients with HbA1c ≥ 8.0%, to allow optimization of glycaemic control. In addition to the short term increased perioperative risks in diabetics, DM is an unrelenting disease with long term sequelae if poorly controlled. High baseline HbA1c value (7.8% ± 1) is associated with diabetes progression over 3 years^32^. Disease progression was defined as advancing to sustained insulin use or HbA1c > 8.5% when treated with two or more OHGAs. For patients with prediabetes, there is also a 5–10% annualized conversion rate to DM, with complications like nephropathy and retinopathy already beginning to develop in the prediabetes state^33^. Conversely, in patients with HbA1c < 6%, the risk of long term complications is reduced^34^. The perioperative period presents a screening opportunity for prediabetes, DM and other chronic medical conditions (e.g.hypertension). For patients with existing conditions, it allows assessment and optimization of those chronic conditions, forming an effective “teachable moment”^35^. Appropriate referrals to other healthcare providers and a multidisciplinary approach would lead to improved long-term outcomes and public health.

In our study, Indian ethnicity was associated with elevated HbA1c levels and poorly controlled DM. In the USA and Europe, even at low BMI, a higher incidence of type 2 DM was reported in Indians compared to other ethnic groups^36,37^. In Singapore, the prevalence of DM in the Indian population was twice as high compared to the Chinese population (20–26% versus 10–13%)^38^. Possible hypotheses for increased DM susceptibility in Indian ethnicity include reduced beta cell function, impaired insulin activity due to low lean mass and ectopic fat deposition on the liver and muscles^39^. There are limited dietary, exercise and behavioural studies conducted among Indian ethnicity and future research into potential clinical and public health interventions to address these susceptibilities are needed.

From the review of medical records, the prevalence of DM in our study population is 19%, higher than the general adult Singapore population (14.2%)^40^. However, this is not unexpected due to the increasing prevalence of DM with age. The mean (SD) age of our study population was 52.1 (16.3) years. Our results are consistent with existing literature on non-cardiac surgical populations which showed similar DM prevalence rates and an older patient population^12,13,41,42^. In the study by Yong et al.^11^ where the participants were aged 55 and above, the prevalence of DM in their population was higher at 30%. Diabetes-related comorbidities may also necessitate surgical intervention, further accounting for the increased prevalence amongst the surgical population.

Undiagnosed diabetics have a three-fold increased risk in 1-year mortality compared to non-diabetics^43^. Early diagnosis, intervention and effective surveillance may reduce the socioeconomic burden on the healthcare system. In our study, the prevalence of undiagnosed DM was surprisingly low at 0.5%. This is markedly reduced from 7.4% reported in an earlier study in 2016^43^. In other international studies, the prevalence ranged from 1.6 to 34%^12, 14,21,41^. Shohat et al. reported a prevalence of 40% in 1461 patients who underwent joint arthroplasty surgery^12^. We postulate that the local take-up rate of the Singaporean government heavily subsidised community diabetes screening programme by 6–7 in every 10 adults aged 40 and above^44^ has contributed to the significant improvement and low rate of undiagnosed DM in our surgical population.

Prior to the recommendation of HbA1c for the diagnosis of DM, fasting blood glucose (FBG) and oral glucose tolerance test (OGTT) had been the established investigations. In the perioperative clinic setting, FBG and OGTT are challenging to perform due to requirements for fasting. The advantages of performing HbA1c testing compared to FBG levels and OGTT include convenience for patients (fasting is not required), pre-analytical stability of the sample and reduced day-to-day variation as a result of stress or illness, reflecting average plasma glucose levels over the previous 8–12 weeks^45^. However, potential drawbacks include HbA1c variability due to the presence of haemoglobin variants (e.g. thalassaemia), ethnicity and conditions that affect red cell turnover (eg. haemodialysis and glucose-6-phosphate-dehydrogenase deficiency)^46^. In view of these limitations, Lim et al. evaluated HbA1c (versus FBG or OGTT) as a diabetes screening modality in a multi-ethnic Singapore population, and demonstrated that HbA1c is an appropriate alternative to FBG^21^. Despite this, we excluded patients with haemoglobinopathies from our analysis as HbA1c assay techniques may be significantly affected by blood samples containing haemoglobin variants^47^.

Conditions that influence erythrocyte turnover may affect HbA1c levels. Anaemia may: (1) increase erythrocyte turnover, lowering HbA1c levels, or (2) reduce turnover or modify configuration of haemoglobin (Hb), and increasing the glycation of its N‐terminal valine, leading to higher HbA1c values^48^. Currently, there is no consensus as to the effect of anaemia on HbA1c^49,50^. Nevertheless, care should be taken while interpreting the results. In our data, we observed no difference in haemoglobin levels in the two groups stratified by HbA1c greater or lower than 6% (Table 1).

This study has several limitations. Firstly, familial history of DM was not elicited in our preoperative assessment. A positive family history of DM^51,52^, especially in first degree relatives, is a strong independent predictor for developing DM, even after accounting for other risk factors (e.g. physical activity, waist circumference and BMI)^53^. Familial history of DM should be considered when conducting targeted preoperative HbA1c screening of surgical patients. Secondly, patients undergoing cardiac surgery (where higher DM prevalence may be present^54^) were excluded and therefore our findings may not accurately reflect the HbA1c distribution among the surgical population. A larger multi-centre study, including all surgical disciplines is required to determine the actual prevalence of DM in the surgical population and to validate a HbA1c screening protocol among elective surgical patients in our local population. Thirdly, this being a prevalence study, it is not sufficiently powered to detect surgical and post-operative complications.

## Conclusion

The prevalence of patients with HbA1c ≥ 6.1% in our study population was 20.8%. Preoperative HbA1C screening identified 4.2% of patients who did not have a previous diagnosis of DM or prediabetes, suggesting that we could utilize the perioperative encounter as a screening opportunity. Targeted preoperative HbA1 screening for at risk elective surgical patients may be more cost-effective and allows focused use of healthcare resources. In line with the ADA recommendations, risk assessment and HbA1c screening should be considered in asymptomatic individuals and if normal, repeated testing at 3 yearly intervals is reasonable.
